# *GBP5* Serves as a Potential Marker to Predict a Favorable Response in Triple-Negative Breast Cancer Patients Receiving a Taxane-Based Chemotherapy

**DOI:** 10.3390/jpm11030197

**Published:** 2021-03-12

**Authors:** Shun-Wen Cheng, Po-Chih Chen, Tzong-Rong Ger, Hui-Wen Chiu, Yuan-Feng Lin

**Affiliations:** 1 Department of Biomedical Engineering, Chung Yuan Christian University, Taoyuan City 32023, Taiwan; g9975606@cycu.edu.tw (S.-W.C.); sunbow@cycu.org.tw (T.-R.G.); 2 Neurology Department, Shuang-Ho Hospital, Taipei Medical University, New Taipei City 235, Taiwan; d620100001@tmu.edu.tw; 3 Taipei Neuroscience Institute, Taipei Medical University, New Taipei City 23561, Taiwan; 4 Department of Neurology, School of Medicine, College of Medicine, Taipei Medical University, Taipei 11031, Taiwan; 5 Graduate Institute of Clinical Medicine, College of Medicine, Taipei Medical University, Taipei 11031, Taiwan; 6 Department of Medical Research, Shuang Ho Hospital, Taipei Medical University, New Taipei City 23561, Taiwan; 7 TMU Research Center of Urology and Kidney, Taipei Medical University, Taipei 11031, Taiwan; 8 Cell Physiology and Molecular Image Research Center, Wan Fang Hospital, Taipei Medical University, Taipei 11031, Taiwan

**Keywords:** triple-negative breast cancer, taxane, chemotherapy, GBP5, Akt/mTOR, autophagy

## Abstract

Pre-operative (neoadjuvant) or post-operative (adjuvant) taxane-based chemotherapy is still commonly used to treat patients with triple-negative breast cancer (TNBC). However, there are still no effective biomarkers used to predict the responsiveness and efficacy of taxane-based chemotherapy in TNBC patients. Here we find that guanylate-binding protein 5 (GBP5), compared to other GBPs, exhibits the strongest prognostic significance in predicting TNBC recurrence and progression. Whereas GBP5 upregulation showed no prognostic significance in non-TNBC patients, a higher GBP5 level predicted a favorable recurrence and progression-free condition in the TNBC cohort. Moreover, we found that GBP5 expression negatively correlated with the 50% inhibitory concentration (IC_50_) of paclitaxel in a panel of TNBC cell lines. The gene knockdown of GBP5 increased the IC_50_ of paclitaxel in the tested TNBC cells. In TNBC patients receiving neoadjuvant or adjuvant chemotherapy, a higher GBP5 level strongly predicted a good responsiveness. Computational simulation by the Gene Set Enrichment Analysis program and cell-based assays demonstrated that GBP5 probably enhances the cytotoxic effectiveness of paclitaxel via activating the Akt/mTOR signaling axis and suppressing autophagy formation in TNBC cells. These findings suggest that GBP5 could be a good biomarker to predict a favorable outcome in TNBC patients who decide to receive a taxane-based neoadjuvant or adjuvant therapy.

## 1. Introduction

Triple-negative breast cancer (TNBC) is a subset of breast cancer that does not express the estrogen receptor (ER), progesterone receptor (PR), and human epidermal growth factor receptor-2 (HER2) [1], and accounts for approximately 20% of breast cancers [2]. TNBC is most aggressive subtype of breast cancers, with a high metastatic ability and lack of specific targeted therapeutics [3]. It has been shown that TNBC patients with the BRCA mutation, higher levels of tumor-infiltrated lymphocytes and p53 abnormalities have a greater pathological complete response (pCR) rate to anthracycline and taxane regimens [4,5,6]. More recently, TNBCs were further classified into six different molecular subtypes—basal-like 1 (BL1), basal-like 2 (BL2), immunomodulatory (IM), mesenchymal stem like (MSL) and luminal androgen receptor (LAR), with a different pathological complete response rate to the standard neoadjuvant regimens include anthracyclines, taxanes, and cyclophosphamide [7]. This classification demonstrated that TNBCs are a heterogeneous group, which explains the lack of survival benefit for experimental drugs tested in several clinical trials. Therefore, identifying useful markers to predict the therapeutic responsiveness in TNBC subtypes is urgently needed in terms of precision oncology.

Guanylate-binding protein 5 (GBP5) has been known as part of the family of interferon-gamma (IFN-γ)-inducible GTPases and is involved in many cellular functions, including inflammasome activation [8] and innate immunity against microbial pathogens [9,10,11,12]. The human GBP family is composed of seven different members (GBP1-7) [13]. In addition to their immunomodulatory functions, a recent report showed that GBP1 upregulation predicts poor prognosis and is probably associated with the mechanism for erlotinib resistance in lung adenocarcinoma [14]. Moreover, GBP1 knockout by the CRISR/Cas9 tool dramatically suppressed the metastatic potential of prostate cancer cells [15]. In ER-negative breast cancer patients with brain metastasis, GBP1 was up-regulated by the stimulation of T lymphocytes, which promoted the ability of breast cancer cells to cross the blood–brain barrier [16]. GBP1 has also been proposed as a potential drug target for treating TNBC with elevated EGFR expression [17]. On the other hand, GBP2 appeared to correlate with favorable prognosis in breast cancer and indicate an efficient T cell response [18]. The methylation of GBP2 promoter was found in TNBC and associated with the malignant evolution of breast cancer [19]. Nevertheless, the prognostic significance of GBP5 and its roles in TNBC development remain largely unknown.

This study thus attempted to estimate the prognostic significance of GBP5 in TNBC patients with systemic chemotherapy. Our results showed that GBP5 upregulation strongly predicts a favorable recurrence and progression-free survival rate in TNBC patients. Particularly, GBP5 upregulation was significantly associated with a pCR rate in breast cancer patients receiving docetaxel/paclitaxel-based neoadjuvant therapy. Cell-based experiments revealed that GBP5 expression is negatively correlated with the 50% inhibitory concentration of paclitaxel in a panel of tested TNBC cell lines. Moreover, our results showed that GBP5 upregulation probably activates the Akt/mTOR pathway and suppresses autophagy formation in the paclitaxel-sensitive TNBC cells. These findings suggest a potential prognostic value of GBP5 in predicting the therapeutic effectiveness of taxane-based regimens in pre and post-operative settings for TNBC patients.

## 2. Materials and Methods

### 2.1. Clinical and Molecular Data for Breast Cancer Patients 

The transcriptional profile generated by RNAseq (polyA þ Illumina HiSeq, Illumina, CA, USA) analysis of the TCGA breast cancer cohort was also downloaded from the UCSC Xena website (UCSC Xena. Available online: http://xena.ucsc.edu/welcome-to-ucsc-xena/, accessed on 1 February 2021). Microarray results with accession numbers GSE36133, GSE21997 and GSE32646 and the related clinical data were obtained from the Gene Expression Omnibus (GEO) database on the NCBI website and Kaplan–Meier Plotter website (http://kmplot.com/analysis/index.php?p=service&cancer=breast, accessed on 1 February 2021). The raw intensities in the .CEL files were normalized by robust multichip analysis (RMA), and fold-change analysis was performed using GeneSpring GX11 (Agilent Technologies, Santa Clara, CA, USA). Relative mRNA expression levels were normalized by the median of all samples and presented as log_2_ values. The gene lists of detected gene sets were obtained from the Molecular Signature Database (https://www.gsea-msigdb.org/gsea/msigdb, accessed on 1 February 2021).

### 2.2. Cell Lines and Cell Culture Condition 

TNBC cell lines MDA-MB-231 and MDA-MB-468 were cultured in Leibovitz’s (L-15) medium (Gibco Life Technologies, Grand Island, NY, USA), supplemented with 10% fetal bovine serum (FBS, Invitrogen, Thermo Fisher Scientific, Waltham, MA, USA), and incubated at 37 °C with free gas exchange with atmospheric air. TNBC cell lines HCC2157, HCC38, HCC1143 and HCC1937 were cultured in RPMI-1640 medium (Gibco Life Technologies, Thermo Fisher Scientific, Waltham, MA, USA) with 10% FBS and incubated at 37 °C with 5% CO2. TNBC cell line Hs578T and embryonic kidney cell line 293T were cultured in DMEM with 10% FBS and incubated at 37 °C with 5% CO2. Human non-malignant mammary epithelial cell lines H184B5F5/M10 and MCF10A were cultivated in Alpha-Minimum essential medium supplemented with 10% FBS and DMEM/F-12 medium supplemented with 5% horse serum, 20 ng/mL epithelium growth factor, 0.5 mg/mL Hydrocortisone, 100 ng/mL cholera toxin, and 10 μg/mL insulin, respectively. All cell lines, except H184B5F5/M10 from Bioresource Collection and Research Center (BCRC) in Taiwan, were obtained from American Type Culture Collection (ATCC). All cells were routinely authenticated on the basis of short tandem repeat (STR) analysis, morphologic and growth characteristics and mycoplasma detection.

### 2.3. Reverse Transcription PCR (RT-PCR) 

The total RNA of detected cells was extracted by using TRIzol extraction kit (Invitrogen, Thermo Fisher Scientific, Waltham, MA, USA). The extracted total RNA (5 μg) were treated with M-MLV reverse transcriptase (Invitrogen) and then amplified by PCR protocol with a Taq-polymerase (Protech, Taipei, Taiwan) using paired primers (for GBP5, forward-GCCATTACGCAACCTGTAGTTGTG and reverse-CATTGTGCAGTAGGTCGATAGCAC; for PD-L1, forward-GCTGCACTTCAGATCACAGATGTG and reverse-GTGTTGATTCTCAGTGTGCTGGTC; for GAPDH, forward-AGGTCGGAGTCAACGGATTTG and reverse-GTGATGGCATGGACTGTGGTC).

### 2.4. MTT Assay 

Cells (1 × 10^5^/mL) were cultivated in a 96-well culture plate. At the endpoint of the designated treatments, 10 μL of MTT (3-(4,5-dimethylthiazol-2-yl)-2,5-diphenyltetrazolium bromide) (Molecular Probe, Invitrogen, CA, USA) stock solution was added into each well. The conversion of MTT to formazan by viable cells was performed at 37 °C for another 4 h. Then, to solubilize the formazan precipitates, 100 μL of DMSO solution was added into each well. The levels of formazan were measured by optical density at 540 nm using an ELISA reader in order to estimate cell survival rates.

### 2.5. Lentivirus-Driven shRNA Infection 

Non-silencing and GBP5 shRNA clones (TRCN0000158813 (sh1): CCGGGCCATAATCTCTTCATTCAGACTCGAGTCTGAATGAAGAGATTATGGCTTTTTTG; TRCN0000159924 (sh2): CCGGCAAGGTAGTGATCAAAGAGTTCTCGAGAACTCTTTGATCACTACCTTGTTTTTTG) with a puromycin selection marker were obtained from the National RNAi Core Facility Platform in Taiwan. Lentiviruses were produced by transfecting 293T cells with the shRNA-expressing vector and pMDG/p△8.91 constructs using a calcium phosphate transfection kit (Invitrogen). After incubation for 48–72 h, the media containing lentiviral particles were collected. Cells with 50% confluence grown on six-well plates were cultivated in fresh media containing 5 μg/mL polybrene (SantaCruz, Dallas, TX, USA) prior to infection overnight with a lentiviral particle-driven control or candidate gene shRNA at 2–10 multiplicity of infection (MOI). Cells were further cultivated in the presence of puromycin (10 μg/mL) for 24 h in order to select cells stably expressing the control or candidate gene shRNA. RT-PCR analysis was used to confirm the efficiency of gene knockdown.

### 2.6. Western Blotting Analysis 

Aliquots of total protein (20–100 μg) from designated experiments and TD-PM10315 TOOLS Pre-Stained Protein Marker (10–315 kDa) (BIOTOOLS Co., Ltd., Taipei, Taiwan) were subjected to SDS-PAGE and then transferred to PVDF membranes. The membranes were then incubated with blocking buffer (5% nonfat milk in TBS containing 0.1% Tween-20) for 2 hours at room temperature prior to incubation with primary antibodies against GBP5, (GeneTex, GTX118635, 1;1000), phosphorylated Akt (Thr308) (Taiclone, #tcea12931, 1:500), Akt (Cell Signaling, #4685, 1:1000), phosphorylated mTOR (Cell Signaling, #2971, 1:1000), mTOR (Cell Signaling, #2983, 1:1000), p62 (Mblintl, #PM045, 1:1000) ATG5 (Cell Signaling, #12994, 1:1000), Beclin-1 (Cell Signaling, #3738, 1:1000), LC3-I/II (Cell Signaling, #4108, 1:1000) or GAPDH (AbFrontier, #LF-PA0212, 1:5000) overnight at 4 °C. After excessive washes, the membranes were incubated with peroxidase-labeled species-specific secondary antibodies for another hour at room temperature. Immunoreactive bands were finally visualized by an enhanced chemiluminescence system (Amersham Bioscience, GE Healthcare, Billerica, MA, USA).

### 2.7. Statistical Analysis 

SPSS 17.0 software (Informer Technologies, Roseau, Dominica) was used to analyze statistical significance. Paired *t*-test was utilized to compare GBP5 gene expression in the TNBC tissues. Pearson’s correlation test was performed to estimate the association among mRNA levels of GBP5, IC50 of paclitaxel/doxorubicin and PI3K_AKT_MTOR/MTORC1 gene sets in the detected samples. Kaplan–Meier analysis and log-rank test were used to evaluate survival probabilities. Student’s *t*-test was used to estimate the statistical significance of GBP5 gene expression in clinical samples. The non-parametric Mann–Whitney U was used to analyze the non-parametric data. *p* values < 0.05 in all analyses were considered statistically significant.

## 3. Results

**Figure 1:**
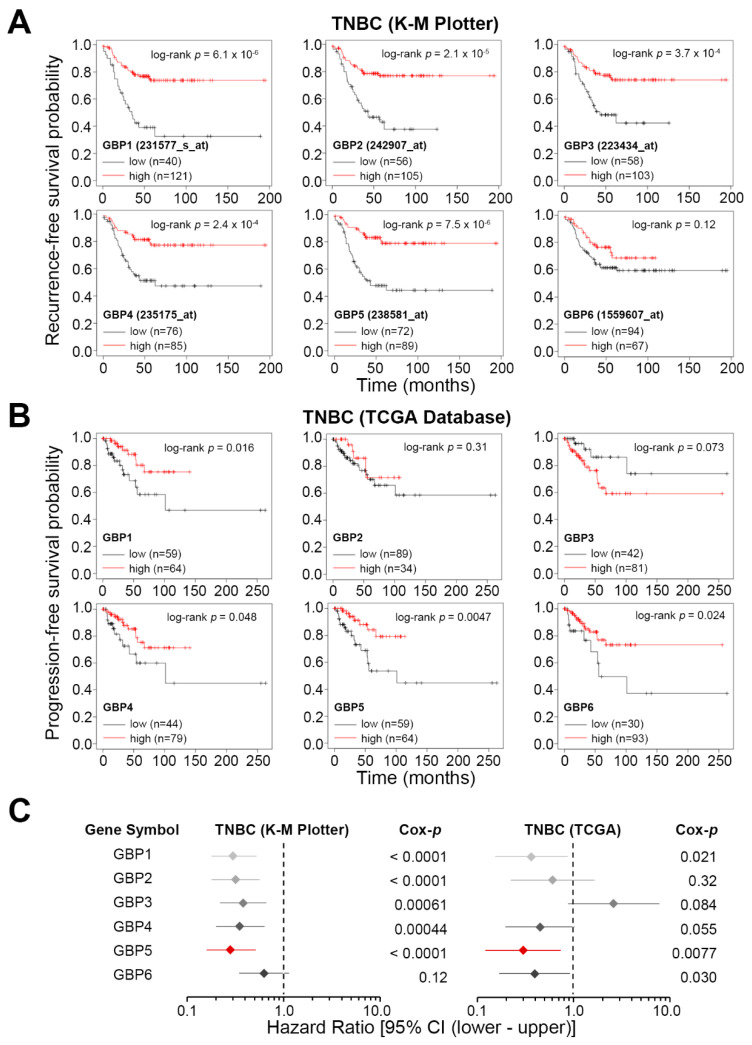
Guanylate-binding protein 5 (GBP5) upregulation predicts a good prognosis in triple-negative breast cancer (TNBC). (A and B) Kaplan–Meier analyses for GBP1, GBP2, GBP3, GBP4, GBP5 and GBP6 gene expression using recurrence-free survival condition against TNBC patients from K–M Plotter (**A**) and progression-free survival condition against TNBC patients from TCGA database (**B**) under a minimized *p* value. (**C**) Forest plot for the hazard ratio at a 95% confidence interval (CI), derived from Cox regression test using univariate mode for GBP1, GBP2, GBP3, GBP4, GBP5 and GBP6 against TNBC cohorts shown in A and B.

**Figure 2:**
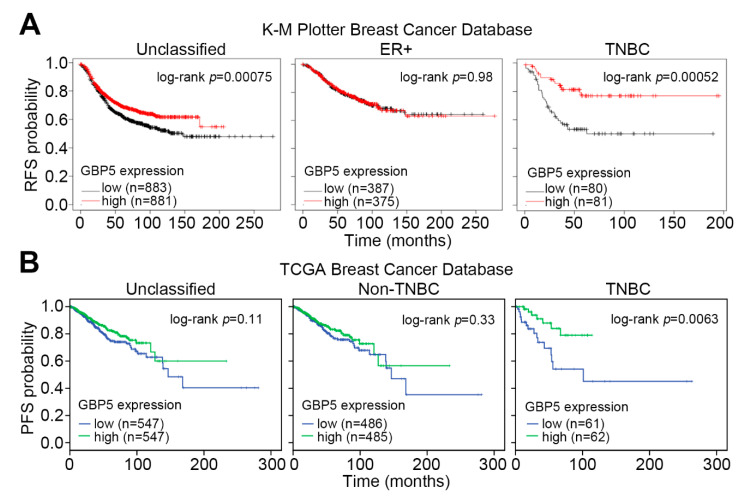
The prognostic significance of GBP5 is dominant for TNBC cohorts. (**A**,**B**) Kaplan–Meier analyses for GBP5 transcripts using recurrence-free for K–M Plotter cohort (**A**) and progression-free for TCGA cohort (**B**) survival conditions against the unclassified (**left**), ER+ or non-TNBC (**middle**), and TNBC (**right**) patients that were stratified by the media of GBP5 mRNA levels.

We first dissected the gene expression status of GBP1, GBP2, GBP3, GBP4, GBP5 and GBP6 in TNBC cohorts stratified into the low and high-risk groups at a minimized log-rank *p* value of Kaplan–Meier analysis, a method determining the optimal cut point in continuous gene expression [20]. In comparison with other GBPs, GBP5 upregulation showed a great correlation with a favorable recurrence-free survival (RFS) rate in TNBC patients from the K–M Plotter (Figure 1A) and progression-free survival (PFS) condition in TNBC patients from the TCGA database (Figure 1B). According to the definition of National Cancer Institute (NCI, https://www.cancer.gov/, accessed on 1 February 2021), RFS and PFS associate with the length of time after primary treatment for a cancer ends that the patient survives without any signs of that cancer and lives with that cancer, but it does not get worse. Both survival conditions could reflect the therapeutic effectiveness in TNBC patients. Moreover, Cox regression test demonstrated that a higher GBP5 level in TNBC patients refers to a favorable hazard ratio, lower than that of other GBPs, under a recurrence and progression-free survival condition for the K–M Plotter and TCGA cohorts, respectively (Figure 1C). Similar views were also found in the other Kaplan–Meier analyses (Figure S1A,B) and Cox regression (Figure S1C) test using overall survival condition. Whereas GBP5 did not show a prognostic significance in the unclassified, ER-positive, non-TNBC population, GBP5 upregulation served as a potential biomarker, predicting a good outcome in TNBC patients under the conditions of recurrence- and progression-free survival probabilities (Figure 2A,B).

**Figure 3:**
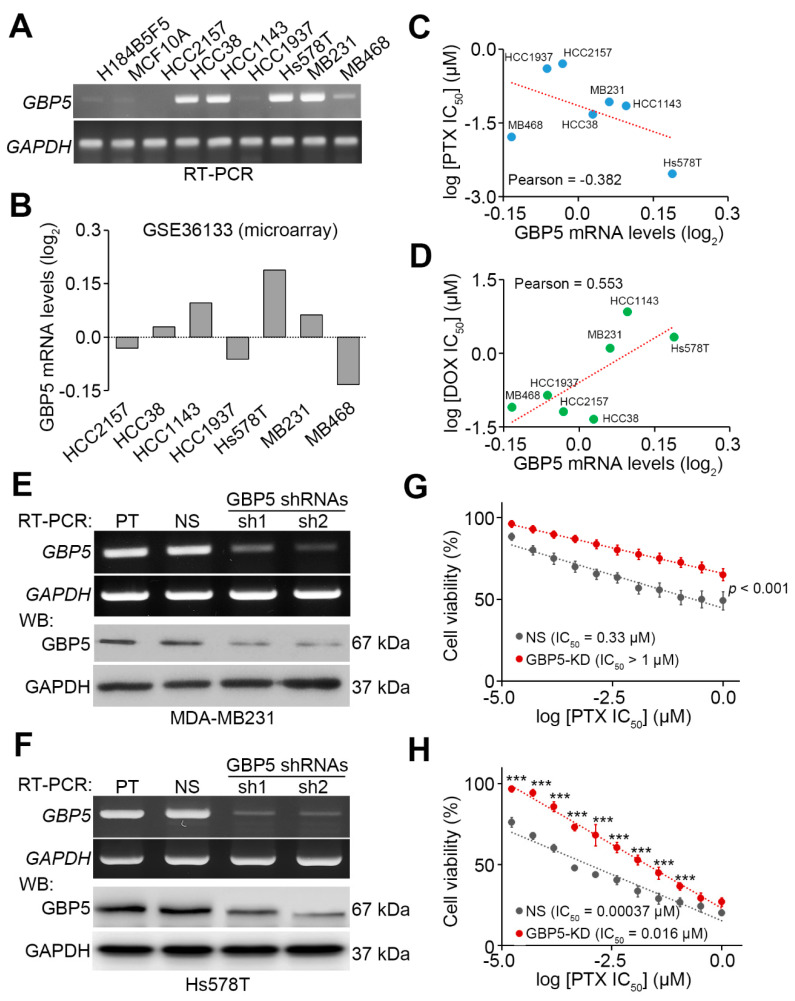
GBP5 knockdown desensitizes TNBC cells to paclitaxel treatment. (**A**) The mRNA levels of GBP5 and GAPDH detected by RT-PCR in a panel of normal mammary epithelial cell lines H184B5F5/M10 and MCF10A, and TNBC cell lines HCC2157, HCC38, HCC1143, HCC1937, Hs578T, MDA-MB231 (MB231) and MDA-MB468 (MB468). (**B**) GBP5 mRNA levels in the indicated TNBC cell lines from GSE36133 dataset. (**C**,**D**) Scatter plots for the correlation of GBP5 mRNA levels with paclitaxel (PTX, **C**) and doxorubicin (DOX, **D**) IC_50_ concentrations in the tested TNBC cells lines. Statistical significance was analyzed by Pearson correlation test. (**E**,**F**) The mRNA and protein levels of GBP5 and GAPDH detected by RT-PCR and Western blot (WB) analyses, respectively, in parental (PT) MDA-MB231 (**E**)/Hs578T (**F**) cells and MDA-MB231/Hs578T cells stably transfected non-silencing (NS) control or 2 independent GBP5 shRNA clones. In **A**, **E** and **F**, GAPDH was used as an internal control of experiments. (**G**,**H**) Dot plot for cell viability determined from non-silencing control and GBP5-knockdown (GBP5-KD), using sh2 clone, MDA-MB231 (**G**)/Hs578T (**H**) cells. Non-parametric Mann–Whitney test was used to estimate the statistical significances. The symbol “***” denotes *p* < 0.001.

We next examined the endogenous mRNA levels of GBP5 in a panel of normal mammary epithelial cell lines H184B5F5/M10 and MCF10A, and TNBC cell lines HCC2157, HCC38, HCC1143, HCC1937, Hs578T, MDA-MB-231 and MDA-MB-468. The data showed that GBP5 mRNA levels in HCC38, HCC1143, Hs578T and MDA-MB-231 cells are much higher than that of HCC2157, HCC1937 and MDA-MB-468 cells, as well as non-malignant H184B5F5/M10 and MCF10A cells (Figure 3A). A similar outcome was also found in the microarray results from GSE36133 dataset for the GBP5 mRNA levels in HCC2157, HCC38, HCC1143, HCC1937, Hs578T, MDA-MB-231 and MDA-MB-468 cells (Figure 3B). WhileGBP5 expression was negatively correlated with the 50% of inhibitory concentration (IC_50_) for paclitaxel (Figure 3C), GBP mRNA levels appeared to be positively correlated with the IC_50_ for doxorubicin (Figure 3D) in those TNBC cell lines. The gene knockdown of GBP5 (Figure 3E,F) by shRNA clone 2 (sh2) which has been validated to suppress GBP5 expression in the previous report [21] predominantly desensitizes MDA-MB-231 and Hs578T cells to the paclitaxel treatment as shown by an increased IC_50_ from 0.33 μM to over 1 μM and 0.00037 μM to 0.016 μM, respectively (Figure 3G,H).

**Figure 4:**
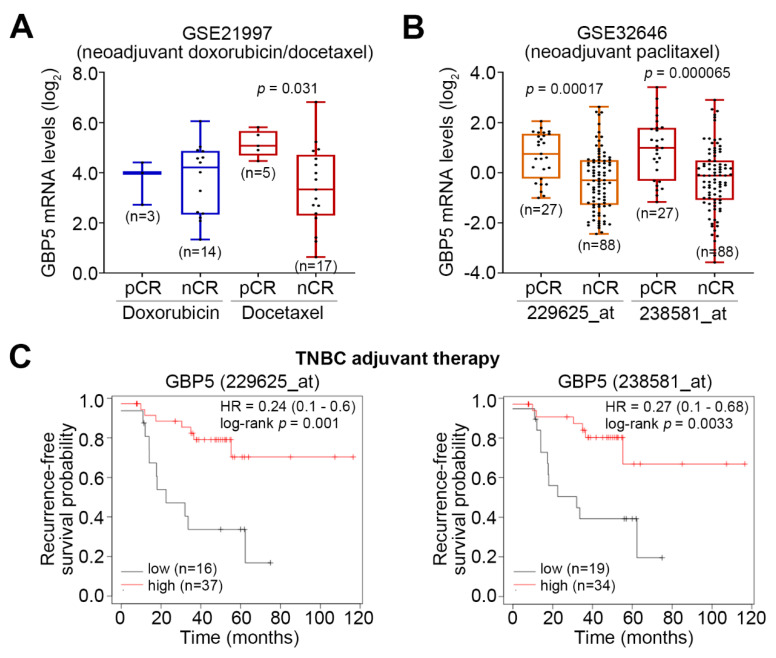
GBP5 upregulation predicts a good responsiveness to the taxol treatment in TNBC patients. (**A**,**B**) Box plots for the GBP5 mRNA levels in breast cancer patients that were recorded to be pathologic complete response (pCR) or no complete response (nCR) after neoadjuvant doxorubicin or docetaxel therapy from GSE21997 dataset (**A**) and after neoadjuvant paclitaxel therapy from GSE32646 dataset (**B**). In B, 229625_at and 238581_at denote the probe identifiers of GBP5 in the commercial microarray. Student’s *t*-test was used to analyze the statistical significance. (**C**) Kaplan–Meier analyses using recurrence-free survival condition for GBP5 mRNA levels detected by two probes in K–M Plotter against TNBC patients receiving adjuvant chemotherapy.

While a higher GBP5 level was probably correlated with no complete response in breast cancer patients received doxorubicin neoadjuvant therapy, GBP5 upregulation appeared to significantly (*p* = 0.031) predict pathologic complete response in breast cancer patients receiving docetaxel neoadjuvant therapy (Figure 4A). Accordingly, in breast cancer cohort received paclitaxel neoadjuvant therapy, GBP5 upregulation significantly (*p* < 0.001) referred to a pathologic complete response (Figure 4B). In the TNBC cohort receiving adjuvant chemotherapy, GBP5 upregulation was robustly correlated with a favorable recurrence-free survival condition (Figure 4C).

**Figure 5:**
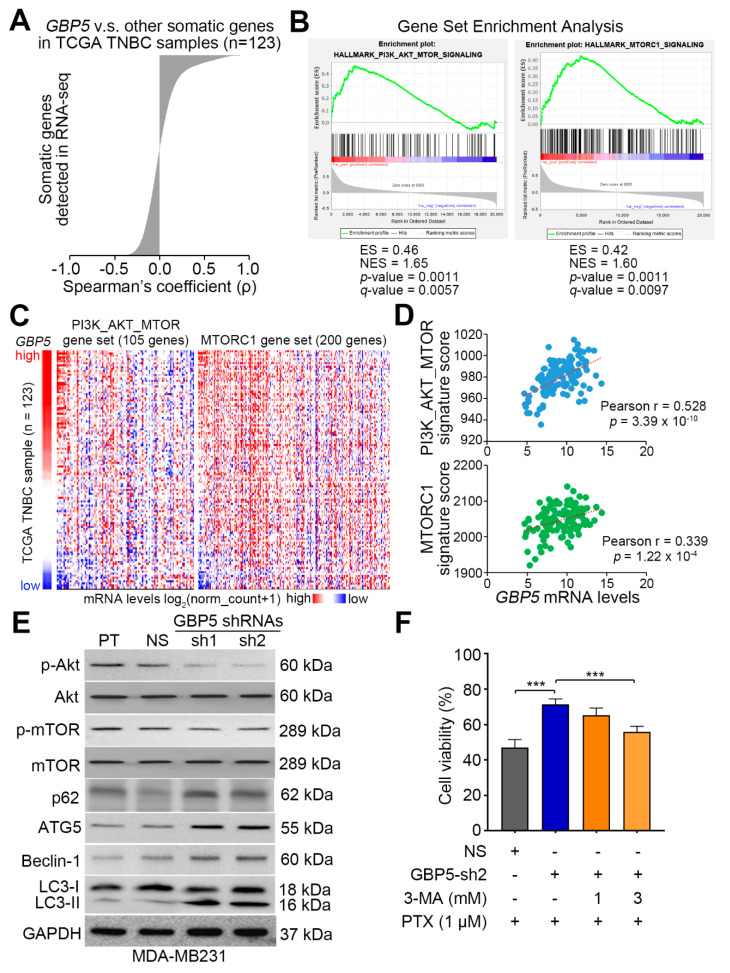
GBP5 activates Akt/mTOR signaling axis and inhibits autophagy activity to the paclitaxel-sensitive TNBC cells. (**A**) A histogram for the Spearman’s coefficient (*p*) values derived from the Spearman correlation test against the co-expression of GBP5 with other somatic genes detected by RNA-sequencing method in 123 TNBC samples deposited in TCGA database. (**B**) The enrichment score (ES) derived from the correlation of GBP5 signature with the PI3K_AKT_MTOR (left) and MTORC1 (right) gene sets was plotted as the green curve. The parameters of enrichment score (NES), nominal *p* value and false discovery rate q value are shown as insets. (**C**) Heatmap for the transcriptional profiling of GBP5 and PI3K_AKT_MTOR (left)/MTORC1 (right) gene sets detected by RNA-sequencing tool in TNBC sample from TCGA database. (**D**) Scatchard plot for the expression of GBP5 and PI3K_AKT_MTOR (upper)/MTORC1 (lower) gene sets in the TNBC samples from TCGA database. (**E**) Western blot analyses for the protein levels of phosphorylated Akt (*p*-Akt), Akt, *p*-mTOR, mTOR, p62, ATG5, Beclin-1, LC3-I/II and GAPDH in the indicated cell variants of MDA-MB231 cells. (**F**) A histogram for the cell viability (percentages relative to untreated groups) in the non-silencing control MDA-MB231 cells and GBP5-silencing MDA-MB231 cells pretreated without or with autophagy inhibitor 3-Methyladenine (3-MA) at 1 and 3 mM prior to the treatment with paclitaxel (PTX) at 1 μM for 72 h. Non-parametric Mann–Whitney U test was used to estimate statistical significance. The symbol “***” denotes *p* < 0.001.

To understand the possible mechanism by which GBP5 upregulation enhances the taxane sensitivity of TNBC, we next performed a computational simulation by using Gene Set Enrichment Analysis (GSEA) program. To obtain a GBP5-related signature, we first performed Spearman’s Correlation tests against the co-expression of GBP5 with other somatic genes determined by the RNA-sequencing tool in TNBC samples from the TCGA database. Then, the ranked Spearman’s coefficient *p* values was used as a GBP5-related signature for the further GSEA simulation (Figure 5A). GSEA results revealed that the GBP5 signature positively correlates with the mRNA levels of gene sets for the PI3K_AKT_MTOR and MTORC1 pathways in TNBC (Figure 5B,D). Western blot analyses revealed that GBP5 knockdown, via its two independent shRNA clones, dramatically suppresses the protein levels of phosphorylated Akt and mTOR, but elevates the protein levels of molecules, p62, ATG5, Beclin1 and LC3-II, related to autophagy formation in MDA-MB-231 and Hs578T cells (Figure 5E and Figure S2). The massive accumulation of LC3-II in the GBP5-silencd MDA-MB-231 cells treated with chloroquine indicate a generation of autophagic flux after GBP5 knockdown (Figure S3). Moreover, the pre-treatment with autophagy inhibitor 3-methyladenin (3-MA) dramatically restored the paclitaxel sensitivity of GBP5-sliencing MDA-MB-231 cells (Figure 5F).

**Figure 6:**
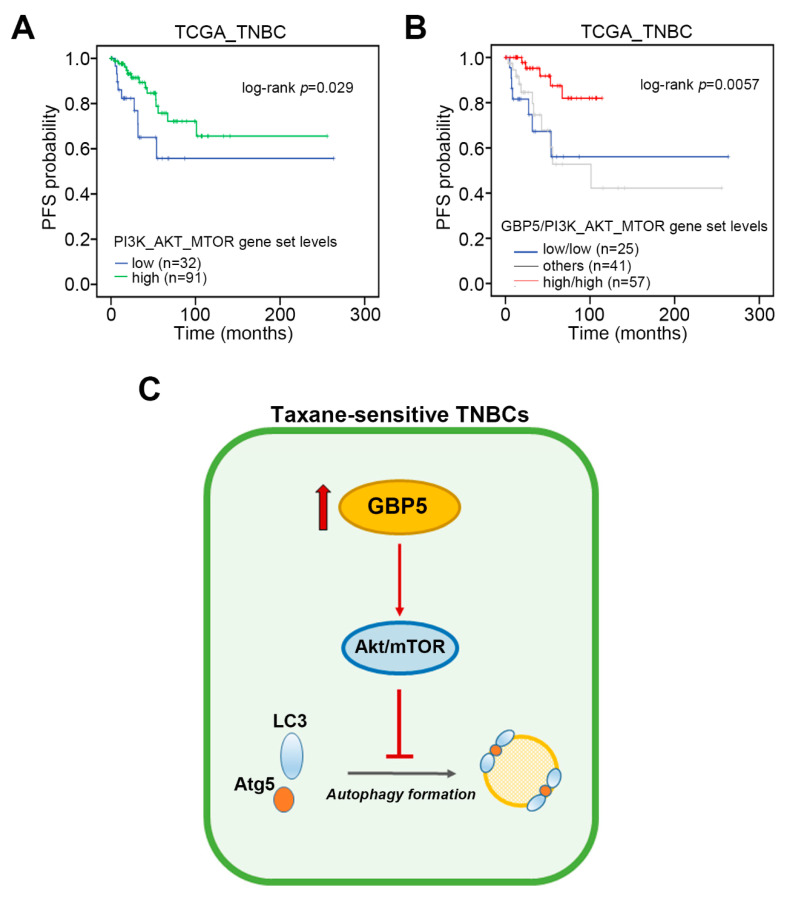
The signature of combining higher levels of GBP5 and PI3K_AKT_MTOR gene set correlates with a favorable progression-free condition in TNBC patients. (**A**,**B**) Kaplan–Meier analyses for the expression of PI3K_AKT_MTOR gene set without (**A**) or with (**B**) the combination of GBP5 expression using progression-free survival condition under a minimized log-rang *p* value against TCGA TNBC patients. (**C**) A possible mechanism for the GBP5-enhanced taxane sensitivity in TNBC.

We further performed Kaplan–Meier analyses using minimize *p* value approach for determining the mRNA levels of PI3K_AKT_MTOR gene set in TNBC patients of TCGA database stratified into low and high-risk groups under progression-free survival condition. The data showed that a higher mRNA level of the PI3K_AKT_MTOR gene set refers to a good progression-free survival condition in TNBC patients (Figure 6A). Importantly, the signature of combining high-level GBP5 and PI3K_AKT_MTOR gene set predicted a prolonged time interval for cancer progression in TNBC patients from the TCGA database (Figure 6B). Collectively, we proposed that GBP5 upregulation probably enhances the activity of Akt/mTOR signaling cascades and suppresses autophagy formation in the paclitaxel-sensitive TNBC cells (Figure 6C).

## 4. Discussion

TNBC remains the breast cancer subtype with the poorest prognosis. Although transcriptional profiling has identified six different TNBC subtypes with sensitivity to therapies, the heterogeneous nature of TNBCs may point to the difficulty in the management of this breast subtype [22]. Therefore, systemic chemotherapy remains the major regimen for treating TNBC in current clinics, even though several targeted agents have been investigated in clinical trials without demonstrating a clear survival benefit [23]. Here, we show that GBP5 upregulation correlates with pathological complete response in TNBC patients who received docetaxel and paclitaxel neoadjuvanttherapy and a favorable recurrence-free survival condition in TNBC patients receiving post-operative systemic chemotherapy. In TNBC cell lines, GBP5 expression was appeared to highly correlate with cellular sensitivity to the cytotoxicity of paclitaxel. Robustly, GBP5 knockdown rendered the tested TNBC cells resistant to paclitaxel treatment. These findings not only highlight a critical role of GBP5 in regulating cellular responsiveness to paclitaxel but also provide GBP5 as a potential marker to predict the great therapeutic effectiveness of paclitaxel on TNBC patients.

Targeting the PI3K/Akt/mTOR signaling axis has been considered to be a promising therapy for the TNBC subtypes, including basal-like 2 (BL2), luminal androgen receptor (LAR), mesenchymal stem-like (MSL) and mesenchymal (M) [7]. The BL2 subtype has been identified to frequently overexpress growth factor receptors, such as epidermal growth factor receptor (EGFR), IGF1R, and myoepithelial markers and commonly exhibit the poorest response to chemotherapy in comparison with other TNBC subtypes [24]. Both MSL and M subtypes were found to highly associate with epithelial–mesenchymal transition and cell motility and frequently harbor a PI3KCA-activating mutations, which provides a therapeutic opportunity for the PI3K/mTOR inhibitor [7]. Although the LAR subtype expresses androgen receptors with sensitivity to an AR antagonist such as bicalutamide, TNBC patients with LAR tumors, compared to other TNBC subtypes, showed a decreased recurrence-free survival time [23]. Furthermore, the PI3K/AKT pathway plays a key role in tumorigenesis and metabolism, survival and proliferation in cancer cells. Previous research has shown that AKT activation by phosphorylation is a good predictor for paclitaxel treatment but a negative predictor for anthracycline-based chemotherapy in breast cancer [25]. Here, we find that the MDA-MB-231 cell line, as well as Hs578T, has been classified as an MSL subtype [23] and expresses enriched GBP5 levels. Moreover, the gene knockdown of GBP5 reduced cellular sensitivity to paclitaxel treatment and suppressed the activity of the Akt/mTOR pathway in MBA-MB-231 cells. These findings not only confirm the need for the Akt/mTOR pathway for the biologic functions of the MSL subtype, but also provide a predictive value of GBP5 for the therapeutic effectiveness of mTOR inhibitor on TNBC patients with MSL subtype.

Basal-like 1 (BL1) subtype has been identified to highly express cell-cycle and DNA-damage-response genes that suggest their great sensitivity to DNA-damaging agents such as platinum [23] and achieve a higher pCR rate in systemic chemotherapy compared to other subtype [24]. In this study, excepting MDA-MB-231 and Hs578T cells, other TNBC cell lines, HCC2157, HCC38, HCC1143, HCC1937 and MDA-MB-468, are of the BL1 subtype and express the endogenous GBP5 transcript at different levels. HCC38 and HCC1143 cells exhibiting higher GBP5 levels displayed a great sensitivity to paclitaxel treatment compared to HCC2157 and HCC1937 cells, which harbor a lower GBP5 expression. Conversely, the endogenous mRNA levels of GBP5 in these TNBC cell lines with BL1 characters appeared to be negatively correlated with the cellular sensitivity to doxorubicin treatment. Despite its lack of significance, breast cancer patients with tumors expressing a higher GBP5 transcript did not show a complete response to doxorubicin neoadjuvant therapy. Therefore, GBP5 may also serve as a potential marker to predict the therapeutic efficacy of DNA-damaging agents in TNBC patients with the BL1 subtype, even though this type shows a great pCR rate after systemic chemotherapy.

Compared to other breast cancer subtypes, TNBC was found to have the highest count of tumor-infiltrated lymphocytes (TILs) [26,27], indicating immune modulation as the new treatment paradigm in TNBC. Although the role of GBP5 in modulating the immune responses between TNBC and TIL needs to be further explored, it has been identified as an interferon-responsive effector [10,28] and was found to promote the activation of NLRP3-dependent inflammatory responses [8]. Cytotoxic drugs have been found to be capable of modifying the tumor microenvironment, thereby inducing dendritic cell activation and cytotoxic T cells [29,30,31], which support the concept that the immunotherapeutic effectiveness may be amplified by chemotherapy [32]. Besides this, it has been found that the induction of the inflammation-related pathway promotes metastatic progression in breast cancer [33,34]. NF-κB is recognized as a key transcription factor in regulating inflammation-related gene expression [35], as well as PD-L1 expression in lung cancer [36], thereby enhancing the metastatic potentials of TNBC [37,38,39]. Therefore, further experiments are needed to explore the role of GBP5-induced activation of NLRP3-dependent inflammatory response in the immunomodulatory capacity of TNBC after systemic chemotherapy.

## 5. Conclusions

Collectively, molecular subtyping provides a new era of precisely managing TNBC patients who decide to receive systemic chemotherapy, or who are probably sensitive to targeted therapies, e.g., Akt/mTOR inhibitors. Although the prognostic significance of *p*-Akt and *p*-mTOR in TNBC patients receiving pre- or post-operative chemotherapy remains controversial according to previous reports [23,40,41,42], in this study, the signature of combining low-level GBP5 with either a high- or low-level transcript of the PI3K/AKT/MOTR geneset predicted a poorer progression-free survival condition in TNBC patients. These findings suggest that GBP5 may serve as a useful biomarker to predict the therapeutic effectiveness of taxane-based chemotherapy on TNBC subtypes. Even in the BL1 subtype, which is highly sensitive to DNA-damaging agents, e.g., doxorubicin, GBP5 expression is able to distinguish an insensitive population. Importantly, this is the first documentation showing that GBP5 shows prognostic significance and is capable of regulating the activity of Akt/mTOR axis and autophagy formation in TNBC.

## Data Availability

Publicly available datasets GSE21997 and GSE32646 were analyzed in this study and can be found here: https://www.ncbi.nlm.nih.gov/geo/, accessed on 1 February 2021.

## Supplementary Materials

The following are available online at https://www.mdpi.com/2075-4426/11/3/197/s1, Figure S1: Prognostic significance for GBPs against TNBC patients derived from K-M Plotter and TCGA cohorts under overall survival condition, Figure S2: Western blot analyses for the protein levels of phosphorylated Akt (p-Akt), Akt, p-mTOR, mTOR, p62, ATG5, Beclin-1, LC3-I/II and GAPDH in the indicated cell variants of Hs578T cells, Figure S3: Western blot analyses for the protein levels of LC3-I/II and GAPDH in the parental/non-silencing control MDA-MB231 cells and GBP5-silencing MDA-MB-231 cells without (untreated, UT) or with chloroquine (CQ) treatment at 20 μM for 24 h, Figure S4: Uncut blots for Figure 3E,F, Figure S5: Uncut blots for Figure 5E, Figure S6: Uncut blots for Figures S2 and S3.
