# Supplementary material for: GBP5 Serves as a Potential Marker to Predict a Favorable Response in Triple-Negative Breast Cancer Patients Receiving a Taxane-Based Chemotherapy
Source: J Pers Med. 2021 Mar 12;11(3):197. doi: 10.3390/jpm11030197 (PMC8001168; doi:10.3390/jpm11030197)
Supplement: Supplementary file 1 [file jpm-11-00197-s001.pdf]

Journal of Personalized Medicine

Supplementary Information

GBP5 Serves as a Potential Marker to Predict a Favorable Response in Triple-Negative Breast Cancer Patients Receiving a Taxane-Based Chemotherapy

Shun-Wen Cheng, Po-Chih Chen, Tzong-Rong Ger, Hui-Wen Chiu* and Yuan-Feng Lin*

Content:

Figure S1: Prognostic significance for GBPs against TNBC patients derived from K-M Plotter and TCGA cohorts under overall survival condition.

Figure S2: Western blot analyses for the protein levels of phosphorylated Akt (p-Akt), Akt, p-mTOR, mTOR, p62, ATG5, Beclin-1, LC3-I/II and GAPDH in the indicated cell variants of Hs578T cells.

Figure S3: Western blot analyses for the protein levels of LC3-I/II and GAPDH in the parental/non-silencing control MDA-MB231 cells and GBP5-silencing MDA-MB-231 cells without (untreated, UT) or with chloroquine (CQ) treatment at 20 µM for 24 hours.

Figure S4: Uncut blots for Figure 3E and 3F.

Figure S5: Uncut blots for Figure 5E.

Figure S6: Uncut blots for Figure S2 and S3.

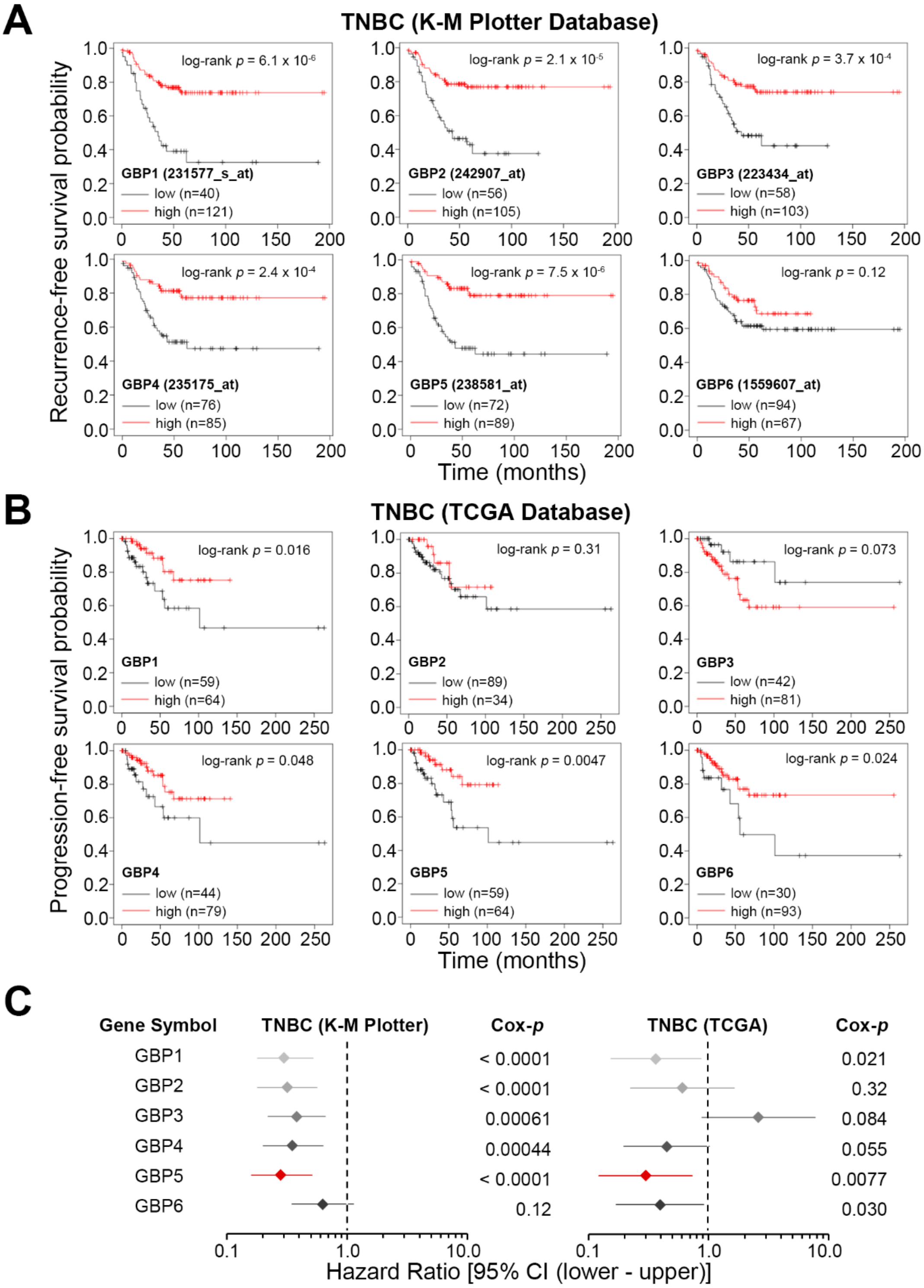

Figure S1: Prognostic significance for GBPs against TNBC patients derived from K-M Plotter and TCGA cohorts under overall survival condition. (A and B) Kaplan-Meier analyses for GBP1, GBP2, GBP3, GBP4, GBP5 and GBP6 gene expression using overall survival condition against TNBC patients from K–-M Plotter (A) and TCGA database (B) under a minimized p value. (C) Forest plot for the hazard ratio at a 95% confidence interval (CI), derived from Cox regression test using univariate mode for GBP1, GBP2, GBP3, GBP4, GBP5 and GBP6 against TNBC cohorts shown in A and B.

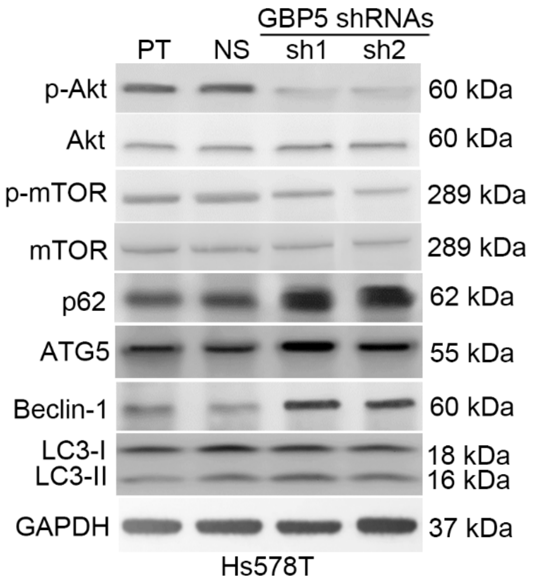

Figure S2: Western blot analyses for the protein levels of phosphorylated Akt (p-Akt), Akt, p-mTOR, mTOR, p62, ATG5, Beclin-1, LC3-I/II and GAPDH in the indicated cell variants of Hs578T cells. GAPDH was used as an internal control of protein loading.

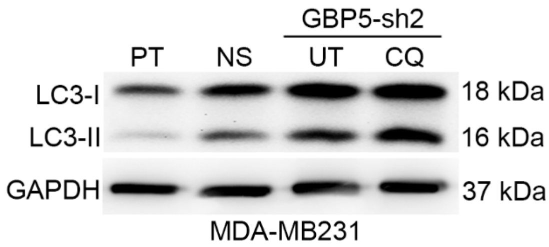

Figure S3: Western blot analyses for the protein levels of LC3-I/II and GAPDH in the parental/non-silencing control MDA-MB231 cells and GBP5-silencing MDA-MB-231 cells without (untreated, UT) or with chloroquine (CQ) treatment at 20 µM for 24 hours. GAPDH was used as an internal control of protein loading.

Figure 3E

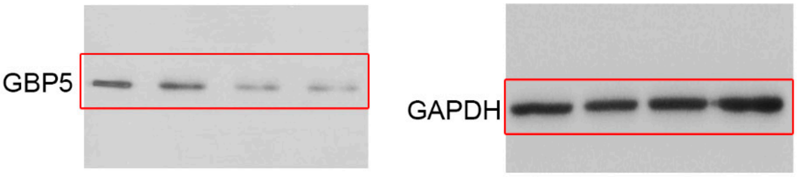

Figure 3F

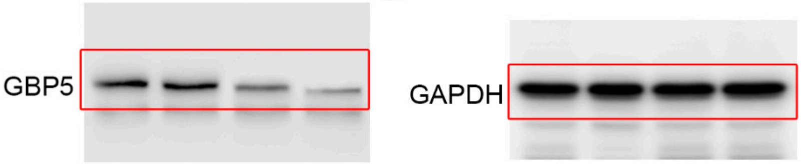

Figure S4: Uncut blots for Figure 3E and 3F.

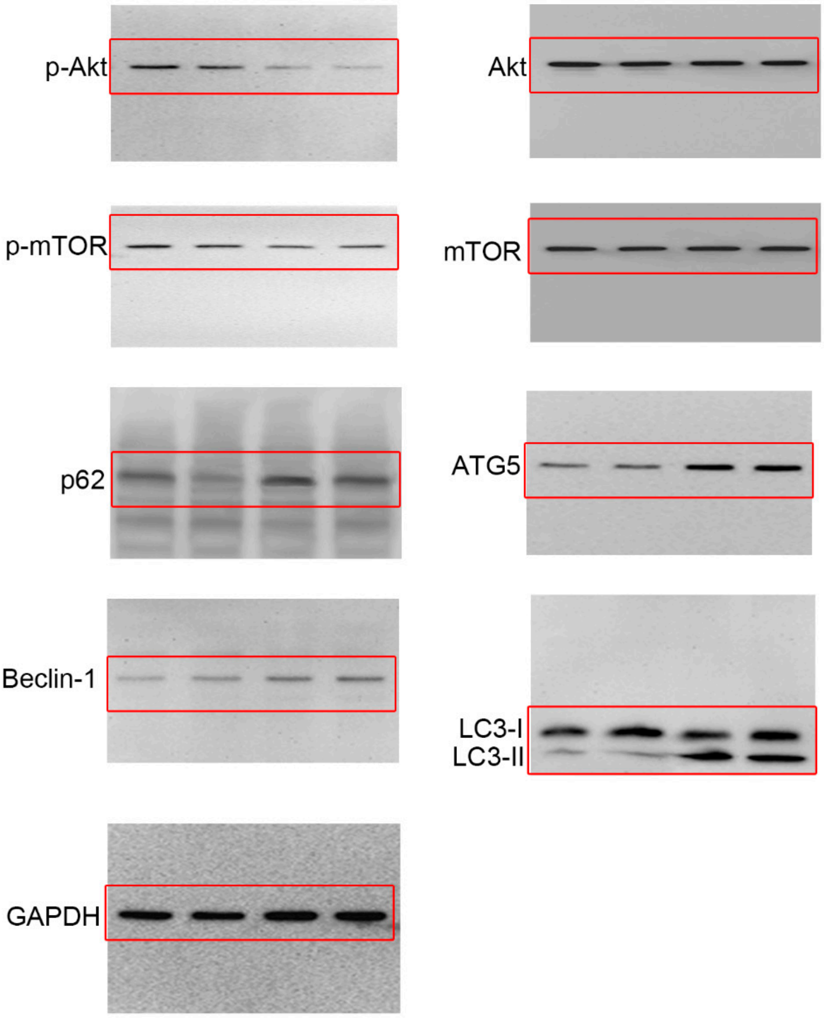

Figure S5: Uncut blots for Figure 5E.

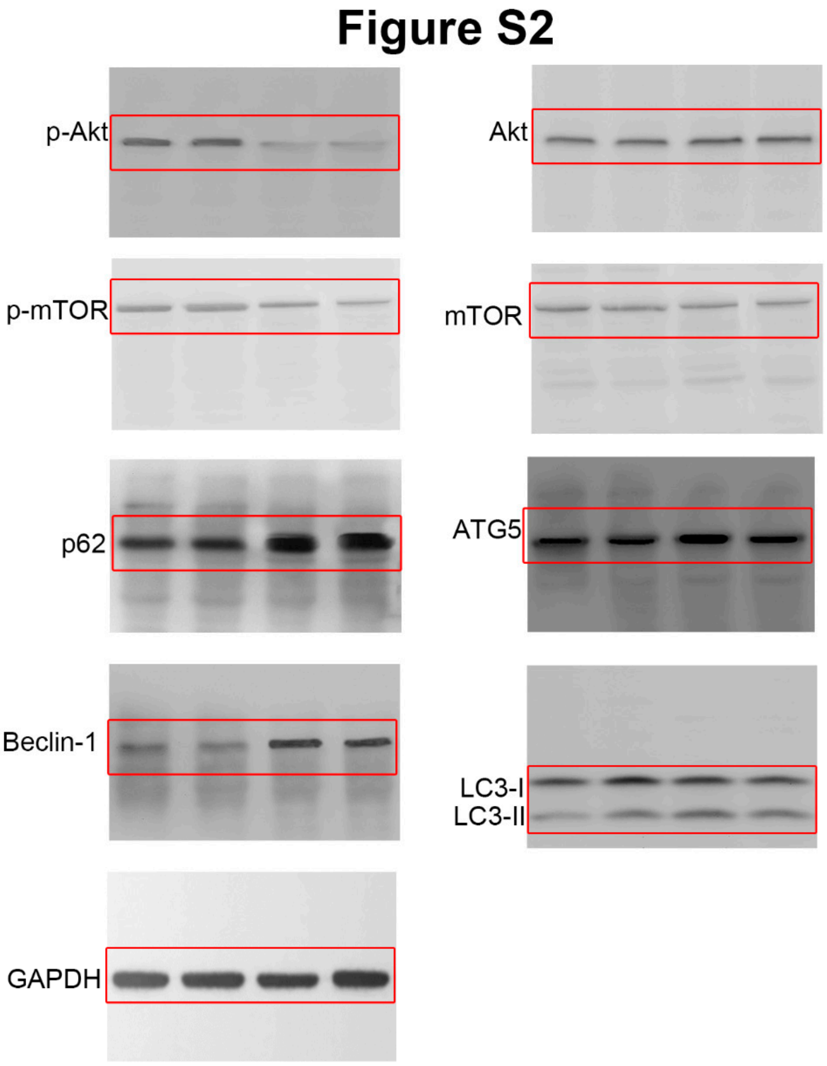

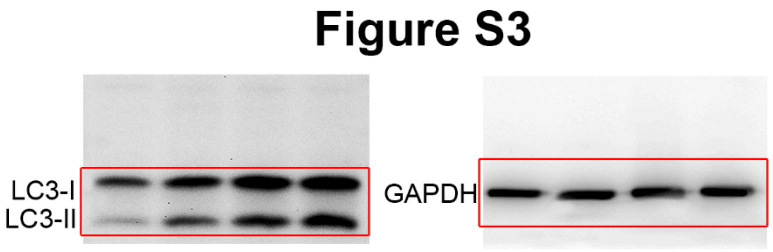

Figure S6: Uncut blots for Figure S2 and S3.
